# Color-Coded Droplets and Microscopic Image Analysis for Multiplexed Antibiotic Susceptibility Testing

**DOI:** 10.3390/bios11080283

**Published:** 2021-08-19

**Authors:** Yunjin Jeong, Haewook Jang, Junwon Kang, Juhong Nam, Kyoungseob Shin, Sunghoon Kwon, Jungil Choi

**Affiliations:** 1 Bio-MAX Institute, Seoul National University, Seoul 08826, Korea; jeancompany@snu.ac.kr; 2 Interdisciplinary Program in Bioengineering, Seoul National University, Seoul 08826, Korea; royjang12@snu.ac.kr (H.J.); jwon225@snu.ac.kr (J.K.); 3 Integrated Major in Innovative Medical Science, Seoul National University, Seoul 03080, Korea; 4 Department of Electrical and Computer Engineering, Seoul National University, Seoul 08826, Korea; njh1103@snu.ac.kr (J.N.); arquina94@snu.ac.kr (K.S.); 5 Institute of Entrepreneurial Bio Convergence, Seoul National University, Seoul 08826, Korea; 6 Biomedical Research Institute, Seoul National University Hospital, Seoul 03080, Korea; 7 Center for Medical Institute, Seoul National University Hospital, Seoul 03080, Korea; 8 School of Mechanical Engineering, Kookmin University, 77 Jeongneung-ro, Seongbuk-gu, Seoul 02707, Korea

**Keywords:** droplet, color code, antibiotic resistance, image processing, multiplexed

## Abstract

Since the discovery of antibiotics, the emergence of antibiotic resistance has become a global issue that is threatening society. In the era of antibiotic resistance, finding the proper antibiotics through antibiotic susceptibility testing (AST) is crucial in clinical settings. However, the current clinical process of AST based on the broth microdilution test has limitations on scalability to expand the number of antibiotics that are tested with various concentrations. Here, we used color-coded droplets to expand the multiplexing of AST regarding the kind and concentration of antibiotics. Color type and density differentiate the kind of antibiotics and concentration, respectively. Microscopic images of a large view field contain numbers of droplets with different testing conditions. Image processing analysis detects each droplet, decodes color codes, and measures the bacterial growth in the droplet. Testing *E. coli* ATCC 25922 with ampicillin, gentamicin, and tetracycline shows that the system can provide a robust and scalable platform for multiplexed AST. Furthermore, the system can be applied to various drug testing systems, which require several different testing conditions.

## 1. Introduction

Antibiotic resistance is an emerging issue in global healthcare [1,2]. Misuse and overuse of antibiotics are the primary factors that are increasing the development of antibiotic-resistant bacteria [3]. Governmental regulation of both antibiotic use and the development of new antibiotics by pharmaceutical companies have been required to address the antibiotic resistance problem. In the clinical environment, determining the antibiotic resistance of bacteria from infected patients, through a process referred to as the antibiotic susceptibility test (AST), is an urgent need to save patients. Current gold standard methods of AST are broth dilution and disk diffusion tests [4]. In the broth dilution test, bacterial samples are loaded into micro-well plates that contain antibiotics and culture media [5]. The micro-well plate is then incubated at 37 °C for 16–20 h, and the optical density of the testing well is measured to determine bacterial growth. Based on the minimal inhibitory concentration (MIC), antibiotic resistance is then determined and the antibiotics needed are prescribed to the infected patient. This gold standard method is considered accurate and applicable in clinical settings. However, there are two limitations of this gold standard method. One is the long incubation time required to differentiate the growth of bacteria in the testing well due to optical density measurement. The other is the scalability of the system, as testing the number of antibiotics and their concentration requires directly increasing the testing well, and the handling process requires a larger system. To address the first limit, the limitation of time, many researchers have developed rapid AST systems. First, single-cell image analysis in microfluidic channels has been found to reduce the time of detection of bacterial growth [6,7,8,9,10]. Immobilization in hydrogel, electrophoresis, and trapping narrow microfluidic channels captures bacterial cells in a small view field that is required for single-cell imaging in high magnification [10,11,12]. Other methods for rapid AST include using a cantilever to monitor the behavior of the cell, fluorescent imaging, and rotating microbeads [13,14,15,16]. These various approaches, in addition to reducing AST time, have been successfully demonstrated, and commercial devices have also been made available [17]. In the case of scalability, droplet microfluidics is an appropriate solution. Droplet microfluidics enables high-throughput handling of small-volume liquid from femto- to nanoliter, which is a useful tool for therapeutic drug assays [18]. Up to 20,000 droplets can be generated per second [19]. The droplets, which are produced by high-throughput screening, play a role in separated reaction chambers, such as aliquoted liquid. To use this advantage of the high-throughput generation of reaction chambers, methods to encode droplets are necessary. The encoding methods have been well-developed in the field of encoded microparticles. However, in comparison to methods to encode microparticles, methods to encode droplets are limited due to the free rotation of droplets, free-floating of materials in droplets, and the limited injection of additional materials [20,21]. Nevertheless, encoded droplets have advantages in acting as tiny bioreactors because encoded microparticles can handle reactions of biomolecules with similar functional groups. These groups are attached to the surface of microparticles [22] or limited chemicals and undergo complicated experiments to calculate the absorbed amount of chemicals [23]. The droplet-encoding methods have been developed using fluorescence [24,25,26], color [27], magnetics [28], encapsulated beads [29], and encapsulated DNAs [30,31]. The codes using magnetics beads or DNAs should be accompanied by complicated decoding processes of hardware [31] or software [29]. The fluorescent or color codes have advantages for assays, such as AST, where various concentrations of the same molecules may be tested [32]. The density of the color codes can easily be used to distinguish the concentration of antibiotics, and the concentration of the colors and the matched molecules can easily be changed by altering the inputs of microfluidics. Besides, the color codes derived from food dyes are compatible with the growth of bacteria and can be easily imaged or viewed using microscopy without fluorescence screening.

Here, we propose a system to test AST in microdroplets with color codes in order to provide the scalability of AST. Different colors of droplets and color densities represent the type and concentration of antibiotics, respectively. The microfluidic droplet-generating system produced many droplets and collected them into a single micro-well-provided multiplexed AST. Images from a color charge-coupled device (CCD) in microscopy were analyzed by image processing, which differentiated color codes and measured the bacterial concentration in the droplet. Testing the clinically important pathogen, *E. coli*, with tetracycline, ampicillin, and gentamicin validated the cultured bacteria in the droplet and decoded color code before and after bacterial growth. Based on the results, color-coded droplets could provide a solution to expand the scale of testing numbers in AST.

## 2. Materials and Methods

### 2.1. Design and Fabrication of Microfluidic Chip

A microfluidic chip was designed to produce water-in-oil droplets in a flow-focusing geometry [26,33]. The width and the depth of the channel were both 200 µm. Two inlets and one outlet were designed to generate droplets and handle the generated droplets, respectively (Figure S1). A silicon wafer was patterned with a SU-8 photoresistor (SU-8 2100, Kayaku Advanced Materials, Tokyo, Japan) using photolithography. Polydimethylsiloxane (PDMS, SYLGARD 184, Dow Corning) was poured onto the patterned silicon wafer and thermally polymerized by heat for 20 min at 150 °C. After polymerization, the PDMS polymer was peeled off of the silicon. The patterned PDMS was bonded to a PDMS-coated glass after being oxidized in air plasma for 2 min (CUTE-MP, Femto Science, Hwaseong, Korea) and heated thereafter for 20 min at 150 °C.

### 2.2. Droplet Generation

Water-in-oil droplets were generated in a flow-focusing geometry of the PDMS microfluidic chip. The inner water of the droplet was a mixture of bacteria, antibiotics, food dye, and bacterial culture media (Figure S2). After mixing with other solutes and solvents, concentrated bacterial culture media with a single bacterial strain were prepared to 10^6^ CFU/mL and 1×, respectively. Food dyes for color codes and antibiotics were first mixed so that color densities would indicate the concentrations of antibiotics. The final concentration of the antibiotics was 0.5, 1, and 2 µg/mL for tetracycline, 0.25 and 1 µg/mL for gentamicin, and 2, 4, and 8 µg/mL for ampicillin. To see if bacteria would grow well in 223–303 µm droplets, control droplets were generated to include only bacteria and culture media without food dye and antibiotics. The outer oil was a mixture of HFE7500 and 1 wt% 008-FluoroSurfactant (RAN Biotechnologies, Beverly, MA, USA). The flow rate of the inner bacterial solution and the outer oil solution were 100 and 1000 µm/h, respectively.

### 2.3. Bacterial Strain Preparation

A standard bacterial strain, *E. coli* ATCC 25922, of the Clinical and Laboratory Standards Institute (CLSI), was purchased from MicroBioLogics, Inc. (Saint Cloud, MN, USA). We conducted all the experiments following the ethical standards from the American Type Culture Collection and performed all the experiments in the biosafety cabinet. In total, 400 µL of a 50% glycerol-deionized water mixture was mixed with 800 µL of a bacterial cation-adjusted Mueller Hinton Broth (CAMHB; BD Biosciences, San Jose, CA, USA) mixture. These stock solutions were placed in microcentrifuge tubes (SPL Life Sciences, Seoul, Korea) and cryopreserved at −70 °C in a deep freezer. For the AST, subcultures were performed before the experiment. Stock solutions from aliquots were inoculated with one loop on Luria–Bertani agar plates (Kisan Bio Co., Ltd., Seoul, Korea) and were incubated in a 37 °C incubator overnight. After 20 to 24 h of incubation, several colonies were formed, and these were used to generate the target concentration of bacteria.

### 2.4. Antibiotics

Gentamicin, ampicillin, and tetracycline were purchased from Sigma-Aldrich (St. Louis, MO, USA). All antibiotics were dissolved in DI water to prepare a stock solution. The antibiotic stock solution was diluted by MHB medium to the target concentration.

### 2.5. Broth Microdilution Test

As a gold standard method of AST, the broth microdilution test (BMD) test was conducted, based on the CLSI’s recommendation, with the purpose of determining the effect of food dye on AST results. Three antibiotics used in this study (ampicillin, gentamicin, and tetracycline) were dissolved in MHB, respectively, and were serially diluted to the desired concentrations from 0.25 to 8 µg/mL. Using a 96-well microtiter plate (BD Biosciences, San Jose, CA, USA), 100 µL of diluted antibiotic solution and 10µL of the bacterial solution were added to each well at a final concentration of 5 × 10^5^ CFU/mL. In an experiment to determine the effect of food dye on AST results, food dye corresponding to each antibiotic was additionally added to each well. The final concentration of each food dye used was consistent with the highest concentration of the food dye used in the color-coded droplet. After 24 h of incubation at 37 °C, an unaided visual inspection determined the results of each microdilution well by comparing the turbidity of the solution with positive control with bacteria and negative control without bacteria.

### 2.6. Microscopic Imaging and Image Processing

**Figure 1:**
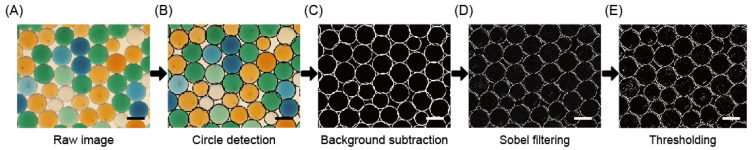
Image analysis processes of color-coded droplets. (**A**) Raw image, (**B**) circle detection, (**C**) background subtraction, (**D**) Sobel filtering, and (**E**) thresholding. Scale bars represent 100 μm.

Microscopy (Nikon ECLIPSE Ti, Tokyo, Japan) with 4× and 10× objective lenses captured the image through a color charge-coupled device (CCD, Nikon Digital Sight DS-Ri1, Tokyo, Japan) of 2048 × 2048 pixels with 1.65 um and 0.66 um, respectively. Several image processing steps were briefly used to obtain color code and bacterial growth data in droplets from the raw CCD images (Figure 1). First, we used the shading compensation method to compensate for the uneven image brightness. Then, to detect the color-coded droplets in the images, we converted the raw images to grayscale and used the Hough transform circle for droplet recognition (Figure 1B). By averaging the color of the rectangular area at the center of the droplet, indicating the length of one side by the radius of each droplet, the color code was analyzed to detect the antibiotics’ type and concentration. We excluded the top and bottom 20% of the pixels based on brightness to minimize noise data. To recognize the bacterial growth in the droplets, we used the Sobel filter, the discrete derivative operator used to obtain image intensity gradient (Figure 1D). High concentrations of bacteria create multiple bacterial swarming patterns in the droplet, resulting in many noise patterns, but are rarely bacteria-free or contain bacteria at low concentrations. Thresholding the Sobel-filtered image determines bacteria growth in droplets through the proportion of areas higher than the threshold in droplets (Figure 1E).

## 3. Results and Discussion

### 3.1. Color-Coded Droplet for Antibiotic Susceptibility Testing

**Figure 2:**
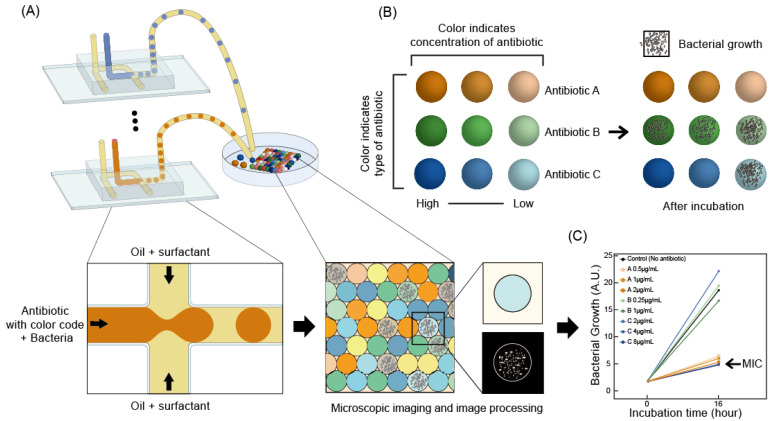
A schematic illustration depicting the creation and analysis of color-coded droplets for antibiotic susceptibility testing. (**A**) Overall processing: generating color-coded droplets, incubating in a single culture well, a microscopic image of the droplet, decoding the color code, and measuring bacterial growth. (**B**) Strategy to generate color code for type and concentration of antibiotics. (**C**) Final determination of MIC values in each antibiotic.

AST determines the minimal inhibitory concentration (MIC) of specific antibiotics to target bacteria, requiring testing with a few serial concentrations of antibiotics. To differentiate the kinds of antibiotics and testing concentration simultaneously, two coding systems are necessary. We used color type and density as coding methods for the antibiotic type and concentration, respectively (Figure 2B). Bacterial samples mixed with antibiotics, color dye, and oil with a surfactant flow into a junction in a cross-shaped microfluidic channel that generates color-coded droplets (Figure 2A). Various color-coded bacterial droplets are mixed in a single well in a micro-well plate and incubated in a 37 °C chamber. If the bacteria are susceptible to antibiotics, it does not increase in number. In other cases, bacteria proliferate, and the microscopic images identify the number of bacteria in droplets. The raw images are analyzed by image processing to decode the type and concentration of antibiotics and measure the bacterial growth. Afterward, the MIC values are derived in the specific antibiotics (Figure 2C).

### 3.2. Measurement of Bacterial Growth in Droplet

**Figure 3:**
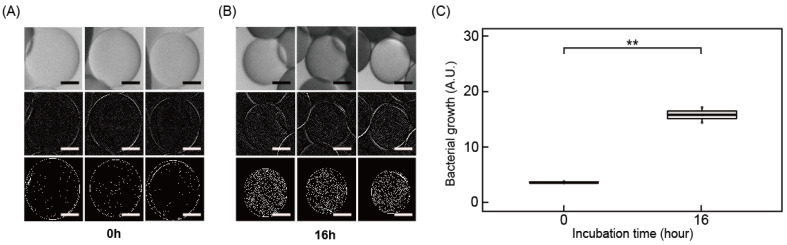
Image analysis of bacterial growth in droplets. Microscopic images of the droplet with bacterial cells at (**A**) the initial condition at a concentration of 10^6^ CFU/mL and (**B**) the final condition after 16 h of incubation. (**C**) Measured bacterial growth of initial and final conditions. Scale bars represent 100 μm in (**A**,**B**).

Measuring the bacterial concentration in the droplet is necessary in order to determine the antibiotic susceptibility of target bacteria. Microscopic imaging of bacterial droplets and following image processing determined the bacterial growth. Previous research on microfluidic AST performed microscopic single-cell analysis to determine bacterial growth. However, in this research, a 10× objective lens was used to take the microscopic image to obtain large numbers of droplets in a large field of view. Therefore, we used a different approach for image analysis. First, the raw image was taken using color CCD, and the edge of the droplet was detected, capturing the inside image of the droplet (Figure 3A,B). After the image was processed, the shadow of the bacterial cell generated different features in the image, and this was transformed into the number of bacteria in the droplet. At the initial point of the droplet, the actual bacterial concentration was 10^6^ CFU/mL, and only a small area was recognized as bacterial cells (Figure 3A,C). After 16 h of incubation, bacterial cells were then detected in bright field images, and the area of bacterial cells from image processing increased as well (Figure 3B,C). Statistical analysis of the droplets at the initial time and 16 h after incubation was based on a *T*-test. When comparing the difference in bacterial growth by selecting three color-coded droplets for each time point, the quantitative analysis between the initial droplet and the incubated droplet showed a significant difference statistically. Here, we set up the imaging time to 16 h of incubation. To reduce detection time, higher magnification of imaging, such as 20× or 40×, is possible.

### 3.3. Differentiation of Color Code of Droplets

**Figure 4:**
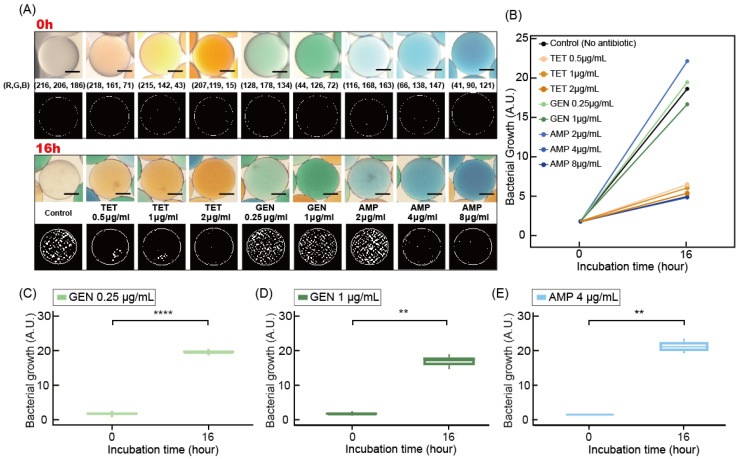
The measurement of bacterial growth in color-coded droplets with antibiotics. (**A**) Microscopic images of the color-coded droplets with different antibiotics and their concentration. Scale bars represent 100 μm. The lower row shows the microscopic images of the color-coded droplet after 16 h of incubation. The binary image shows the bacterial cells from the image processing. (**B**) Graph of bacterial growth at 0 and 16 h of incubation. Statistical analysis of bacterial growth at 0 and 16 h of (**C**) 0.25 µg/mL, (**D**) 1 µg/mL of gentamicin, and (**E**) 4 µg/mL of ampicillin (*p* < 0.001).

To demonstrate that color codes correspond to the type and concentration of antibiotics, we measured the mean of RGB values from each droplet after incubating for 16 h (Figure 4A and Figure S3). All RGB values extracted from droplets in the image were grouped with a similar range (Figure S3). Eighty droplets were randomly selected from a microscope image with a 4× objective lens and clustered. The raw data of RGB values were measured from the inner rectangular of each droplet, of which the length of a side is similar to the radius of the droplet. The final RGB values were then calculated as a mean value of low RGB values after removing the top and bottom 20% values. We coined the final mean value of each color as red, green, and blue, respectively, and the relative luminance was calculated using the following equation:

Relative luminance = 0.2126R + 0.7152G + 0.722B(1)

when clustering with relative luminance values, red, green, and blue values were distinguished into clusters based on 140 and 165, 140 and 105, and 140 relative luminance values, respectively. As a result, the three types and concentrations of antibiotics were successfully classified (Figure 4A). Measuring the bacterial growth of the color-coded droplet was performed by an identical process as the control droplet of no antibiotics, as mentioned in Section 3.3 (Figure 4B). For example, the bacterial growth curves were measured from initial to final state at the cases of 0.25 and 1.0 µg/mL of gentamicin and 4 µg/mL of ampicillin (Figure 4C–E). In all cases, the measured bacterial growth between the initial and final states showed significantly different results. The data validated the feasibility of image processing that measures bacterial growth within the color-coded droplets.

### 3.4. Differentiating Color Code of Droplets

**Figure 5:**
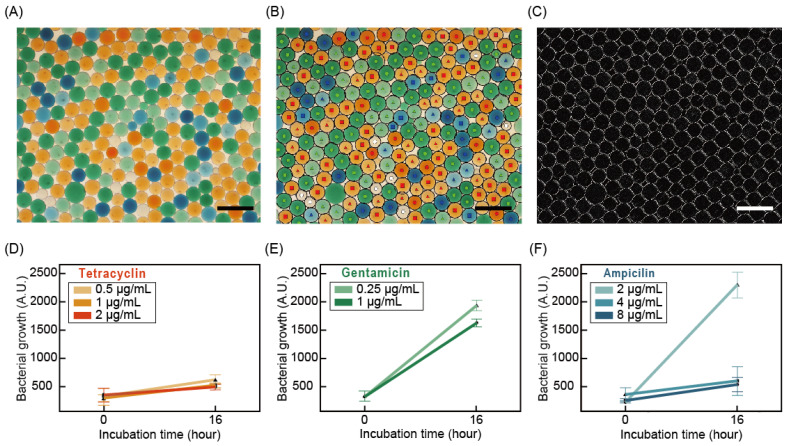
Multiplexed color-coded droplets for antibiotic susceptibility testing. (**A**) Microscopy images of the color-coded multiplexed droplet with bacterial cells. (**B**) Decoding color codes of droplets. ○: control, ▲ (low) ■ (medium) ● (high) concentration of tetracycline, ▲ (low) ■ (medium) concentration of gentamicin, ▲ (low) ■ (medium) ● (high) concentration of ampicillin. (**C**) The detected edges of the droplets. Differences in bacterial growth and interpretation of MIC results with a time of incubation of (**D**) tetracycline, (**E**) gentamicin, and (**F**) ampicillin (standard deviations are indicated). Scale bars represent 500 μm in subfigures (**A**–**C**).

For the multiplexed AST, color-coded droplets of three antibiotics with three concentrations were generated through the method mentioned before. Before the application of color-coded droplets on AST, BMD tests with and without food dye in the testing well showed no significant effect of food dye on the AST (Figure S4). All droplets with different testing conditions were collected in a single well of the micro-well plate. Microscopy captured the images of multiplexed droplets at the initial time and after incubation for 16 h at 37 °C (Figure 5A). In the image, around 200 droplets were captured, including all cases of testing conditions. Image processing detected the droplets’ edges, decoded the color codes of the droplet, and differentiated them (Figure 5B,C). In the case of tetracycline with red color, in all concentrations, no growth of bacteria concluded the MIC value as less than or equal to 0.5 µg/mL. However, gentamicin with a green color code shows growth in all concentrations, concluding the MIC value as greater than 1 µg/mL. In ampicillin with a blue color, at 2 µg/mL, bacteria showed growth after 16 h. However, at 4 and 8 µg/mL, no growth of bacteria was observed. Therefore, the MIC value of ampicillin was determined as 4 µg/mL. In all cases, the difference in bacterial concentration between initial and incubated cases is significant. These data show that using color-code droplets equipped with image processing is capable of multiplexed AST.

## 4. Conclusions

This research proposed color-coded droplets for the multiplexed AST platform. Different colors and their densities were able to differentiate the antibiotic type and its concentration, respectively. Image processing detected the droplet, differentiated the color code, and measured the bacterial growth. A sample test of *E. coli* with three clinically important antibiotics and three concentrations validated the system’s feasibility. Combining various color dyes and their concentrations could expand the number of testing conditions; however, the limitation to our study was that only three antibiotics and concentrations were tested. In addition, when this method is applied to AST, which requires many kinds and concentrations of antibiotics, a long period of time is necessary in order to generate many kinds of droplets. It is important to conduct further studies using droplet microfluidic technologies that can change the concentrations of antibiotics easily and in a timely manner [32,34]. In addition, a microfluidic flow control platform, such as serial dilution, could enhance the system’s scalability.

## Data Availability

Not applicable.

## Supplementary Materials

The following is available online at https://www.mdpi.com/article/10.3390/bios11080283/s1, Figure S1: Design of a microfluidic chip to generate droplets. Figure S2: Preparation of inner water solution to generate bacterial droplets for AST. Figure S3: Histograms represent many droplets at each luminance. Figure S4: BMD test for validation of the effect of food dyes on the results of AST.
