# Supplementary material for: Color-Coded Droplets and Microscopic Image Analysis for Multiplexed Antibiotic Susceptibility Testing
Source: Biosensors (Basel). 2021 Aug 19;11(8):283. doi: 10.3390/bios11080283 (PMC8393621; doi:10.3390/bios11080283)

Supplementary Materials

Color-coded droplets and microscopic image analysis for multiplexed antibiotic susceptibility testing

Yunjin Jeong[1,†], Haewook Jang[2,†], Junwon Kang[2,3,†], Juhong Nam[4], Kyoungseob Shin[4], Sunghoon Kwon[1,2,4,5,6,7,*], Jungil Choi[8,*]

Supplementary Figure 1. Design of a microfluidic chip to generate droplets. The microfluidic chip was designed to generate droplets using a flow-focusing method. The channel width was 200 μm, and the depth of the channel was also designed to be 200 μm. The diameter of the two large circles was 1 mm. The circles were to connect the microfluidic channel and the tubes to inject input solutions perfectly. Two inlets and an outlet were connected to channels at the noted position.

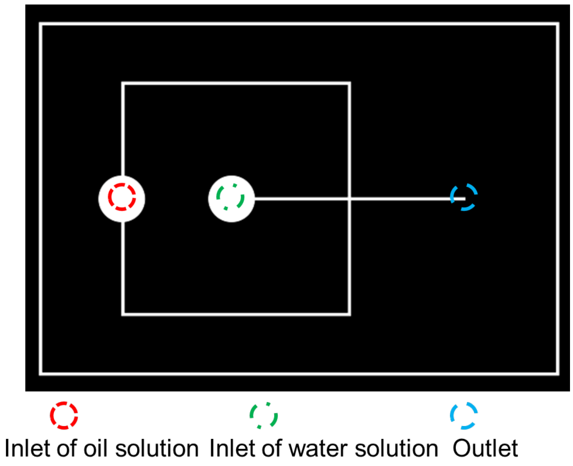

Supplementary Figure 2. Preparation of inner water solution to generate bacterial droplets for AST. Water solutions for droplets were prepared using red, green, or blue food dyes, antibiotics, bacteria, and culture media. The concentration of bacteria and culture media was determined constant for all droplets. However, the concentration of food dyes and antibiotics were variable and proportional to each other. Therefore, food dyes and antibiotics are first mixed and diluted with DI water regarding target concentrations of antibiotics and food dyes.

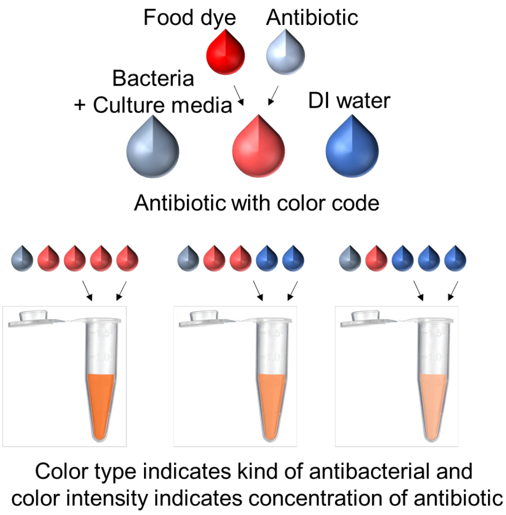

Supplementary Figure 3. Histograms represent many droplets at each luminance. Luminance was calculated by converting from the average RGB value inside the droplet. Two concentrations of gentamicin (A) and three concentrations of tetracycline (B) and ampicillin (C) can be successfully classified by the average luminance of the droplets.

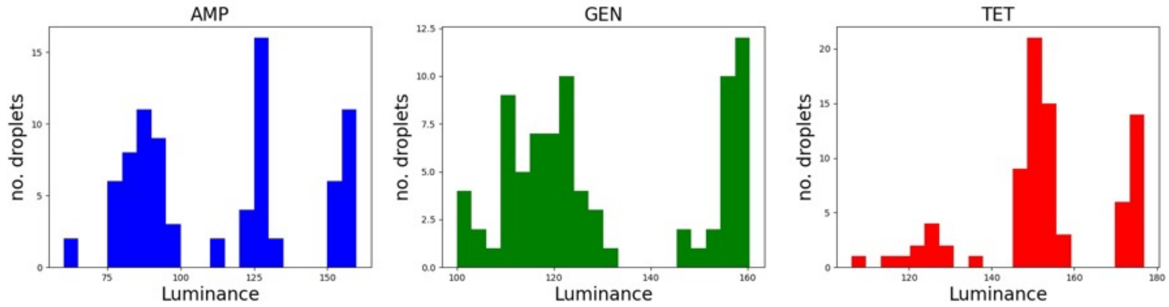

Supplementary Figure 4. BMD test for validation of the effect of food dyes on the results of AST. Results of BMD test (A) without food dyes and (B) with food dyes.

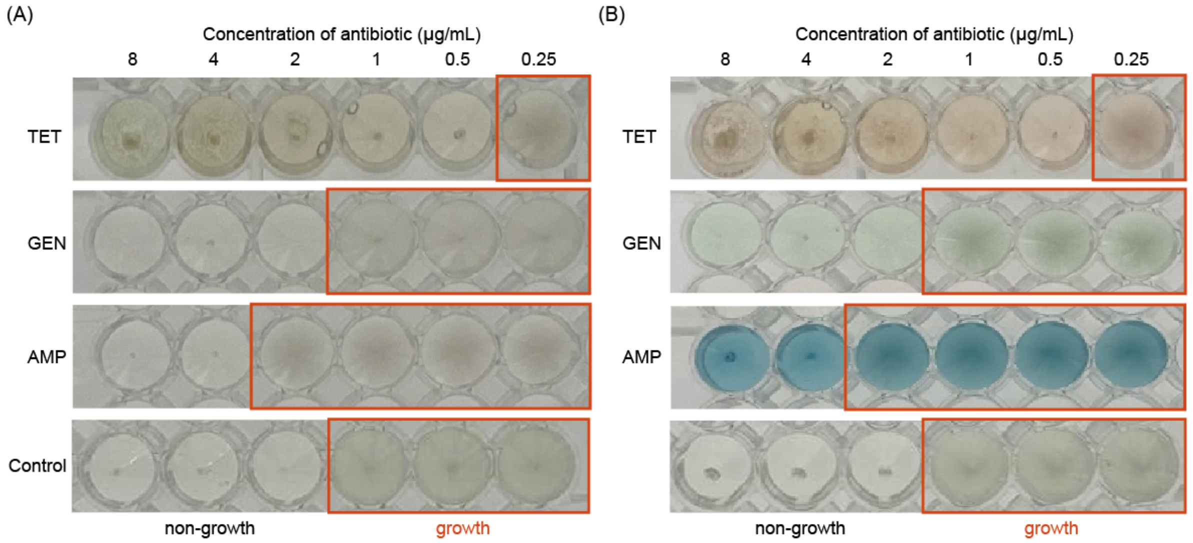

Supplement: Supplementary file 1 [file biosensors-11-00283-s001.zip › biosensors-1348493-supplementary.pdf]
